# Dual Effect of Phenolic Nectar on Three Floral Visitors of *Elsholtzia rugulosa* (Lamiaceae) in SW China

**DOI:** 10.1371/journal.pone.0154381

**Published:** 2016-04-22

**Authors:** Feng-Ping Zhang, Qiu-Yun Yang, Shi-Bao Zhang

**Affiliations:** 1 Key Laboratory of Economic Plants and Biotechnology, Kunming Institute of Botany, Chinese Academy of Sciences, Kunming 650201, Yunnan, China; 2 Yunnan Key Laboratory for Research and Development of Wild Plant Resources, Kunming Institute of Botany, Chinese Academy of Sciences, Kunming 650201, Yunnan, China; University of Cologne, GERMANY

## Abstract

Some plants secrete toxic nectar to appeal to most effective pollinators and deter non-pollinators or nectar thieves; however available information about ecological function of toxic nectar remains scarce. *Elsholtzia rugulosa* stands out as a plant with toxic nectar recorded in SW China. We focused on the functional significance of the phenolic compound that imparts toxic to the nectar of *E*. *rugulosa*. The effects of phenolic nectar were studied in three visitors of the flowers of the winter-blooming *E*. *rugulosa* Hemsl. (Lamiaceae) in SW China. The pollinating species *Apis cerana* Fabricius (Apidae; Asian honey bee) and two occasional visitors, *Vespa velutina* Lepeletier (Vespidae; yellow-legged Asian hornet) and *Bombus eximius* Smith (Apidae; a bumble bee) were tested for their preferences for low and high concentrations of 4-hydroxybenzoic acid in hexose and sucrose solutions. The pollinator is important for the plant, which is dependent on pollinator visits to attain a higher seed production and it is most likely that the combination of phenolic toxic nectar and the adaptation to phenolic nectar by *A*. *cerana* delivers an evolutionary advantage to both actors. The low and high concentrations of the phenolic acid were nearly totally refused by both occasional visitors *V*. *velutina* and *B*. *eximius* and were preferred by the pollinator *A*. *cerana*. *E*. *rugulosa* gains by having a much higher seed production and the pollinating honey bee by having an exclusive and reliable food source during the winter season at high altitudes in SW China. We found that the function of the toxic phenolic compound has dual roles by appealing to legitimate pollinators and deterring non-pollinators of *E*. *rugulosa*.

## Introduction

The function of secondary compounds in plant-animal interactions is one of the intriguing subjects in biological sciences. Secondary compounds such as phenolics (or phenolic acids) are common in floral nectar [1–4]. Phenolics are flavonoid antioxidants characteristic of various honeys and present in the nectar of *Elsholtzia rugulosa* [5]. Nectar containing phenolics deters several flower visitors, including Western honeybee *(Apis mellifera)* [6], and it is estimated that more than 30% of plant species secret phenolic nectar [2]. Some studies have suggested that the presence of phenolics in nectar could play a role in attracting effective pollinators and in deterring undesirable visitors such as generalist pollinators [7–9] and nectar robbers [6, 10], thus reinforcing pollinator fidelity. So far, there is no clear support for this hypothesis and the actual toxicity of the phenolics in the nectar of *Elsholtzia rugulose* has not been proven for its main pollinator, the Asian honey bee. In addition, the effect of pollination by the Asian honey bee on the seed production is unknown. The actual phenolic components of the nectar of *E*. *rugulosa* remain unknown; for the experiments 4-hydroxybenzoic acid was used because it is a well-known phenolic present in honey [11–13], and we refer to the other study which also use this product as a common plant phenolic to test the impact of phenolics on the attraction of the Asian honey bee [14]. Asian honey bee (*Apis cerana*) prefers phenolic-laced sugar solutions over pure sugar solutions, but it is unknown whether this plays any role in the pollination of *Elsholtzia rugulosa* [14, 15]. Moreover, little is known about the reproduction system of this medicinal plant (e.g. Whether *E*. *rugulosa* depends on pollinators for its reproduction, and the fruit set and seed set, etc.). In SW China *E*. *rugulosa* is an important nectar plant at 1300–2400 m, because of its many long-lived flowers during the winter season. No alternative flowers are present and the Asian honey bee depends on the nectar of this plant to overwinter [5]. Neither the significance of the deterrent effect caused by phenolic nectar nor the reproduction system of *E*. *rugulosa* has been investigated previously, although some studies indicate that phenolic compounds increase the flower visitation rate of *Apis cerana* [5, 14].

In this study, we address the following questions: (1) Does *E*. *rugulosa* depends on pollinators for its reproduction, and if so, which of the flower visitors are effective pollinators? (2) Are the effective pollinators attracted by phenolic nectar? (3) Are other floral visitors deterred by phenolic nectar?

## Materials and Methods

The uses of experimental materials were permitted for scientific research by both Ailao Mountain Station for Subtropical Forest Ecosystem Studies and Ailao Mountain National Nature Reserve. No species under first-class state protection were used in this research.

### Study species and sites

*Elsholtzia rugulosa* is an herb to subshrub of 30–150 cm height, which grows at altitudes between 1300 and 2800 m in south-western China. It is an important nectar plant flowering during the winter months of October till December. A natural population of about 300 flowering plants was studied in Ailaoshan, Jingdong, western Yunnan, SW China (geographical coordinates N 24°30’55.64”, E 101°00’38.98”) during October 2013 till December 2014.

### Flower traits

Ten plants were randomly selected in the population for morphological measurements, including the number of inflorescences per plant, flower length and style length, and for measuring the floral lifespan. The standing crop of nectar was sampled from three flowers on each of the 10 plants at 09:30 and 14:30 hr. Nectar volume and sugar concentrations were determined using micro-capillaries and a pocket refractometer (Eclipse 45–81; Bellingham and Stanley Ltd, Tunbridge Wells, Kent, UK).

### Pollinator observations and pollination experiments

Floral visitors were observed between 9:30–17:30 during six successive days (Temperature (°C): 21.88 ± 4.09, relative humidity (%): 55.38 ± 3.36, Mean ± SD). All insect visitors were identified, and their behaviour was recorded. The presence of pollen grains was confirmed on insects (*n* = 35) captured in nets, by collecting pollen samples from their bodies and comparing these to a reference pollen collection. Four pollination treatments were conducted in the study site in October 2014: (1) open pollination: flowers were not manipulated (*n* = 500); (2) bagged control: floral buds were bagged with fine-mesh bags throughout their flowering period (*n* = 480); (3) hand self-pollination: bagged mature flowers were hand-pollinated with pollen from the same inflorescence or the same plant (*n* = 386); (4) hand cross-pollination: bagged flowers were emasculated before anthesis and mature flowers were pollinated with pollen from plants growing 10 m away (*n* = 501). Mature fruits and seeds were collected and counted.

### Behavioral experiments

The 4-hydroxybenzoic acid is a common phenolic compound in many plants, such as buckwheat, cucumber and carrot, etc. [16–18]. The 4-hydroxybenzoic acid of buckwheat honey has a high antioxidant capacity [11–13]. And we refer to the earlier reference which also use this product as a common plant phenolic to test the impact of phenolics on the attraction of the Asian honey bee [14]. We used the 4-hydroxybenzoic acid for behavioral experiments described here, and its concentrations (30 mg phenolics/100 g syrup or 80 mg phenolics/100 g syrup, which are within the concentration range in honey) were used according to the other studies [11, 14].

To assess whether phenolics function in both attraction and deterrence, *Apis cerana* (*n* = 21), and both occasional floral visitors *Vespa velutina* (*n* = 15) and one species *Bombus eximius* (*n* = 13) were captured and kept in individual containers (20 cm^2^) for up to 20 minutes prior to the start of the behavioural experiments, both experiments were conducted on the same bees. They were then offered pure sugar solutions, sugar solutions with low amount of 4-hydroxybenzoic acid as phenolic, and sugar solutions with high amount of 4-hydroxybenzoic acid in the form of 5μL droplets on a 5cm diameter white plastic disk [19]. Two sets of experiments were conducted: (1) They were offered a choice between a 5μL droplet of a hexose solution (20%, w/w) (glucose and fructose in a 1:1 mixture), a 5μL droplet of a 20% hexose solution with 30mg phenolics / 100g hexose solution (20%), and a 5μL droplet of a 20% hexose solution with 80mg phenolics / 100g hexose solution (20%); (2) Same experiment but with hexose replaced by sucrose. Each trial was terminated after an individual bee had probed all three droplets on the disk. The volume of each droplet that remained at the end of the experiment was determined using a calibrated micropipette after feeding for each bee, and the identity and behaviour of individual bees were recorded in each trial.

### Statistical analysis

Difference of fruit set and the number of seeds between different pollination treatments was determined by LSD multiple comparisons test. Kruskal-Wallis tests were used to analyse the preference trials. The fruit set in pollination experiments was compared using an ANOVA. All analyses were performed using SPSS 16 (SPSS Company, Chicago, Illinois, USA), with measured variables presented as mean ± SE.

## Results

### Flower traits

**Fig 1:**
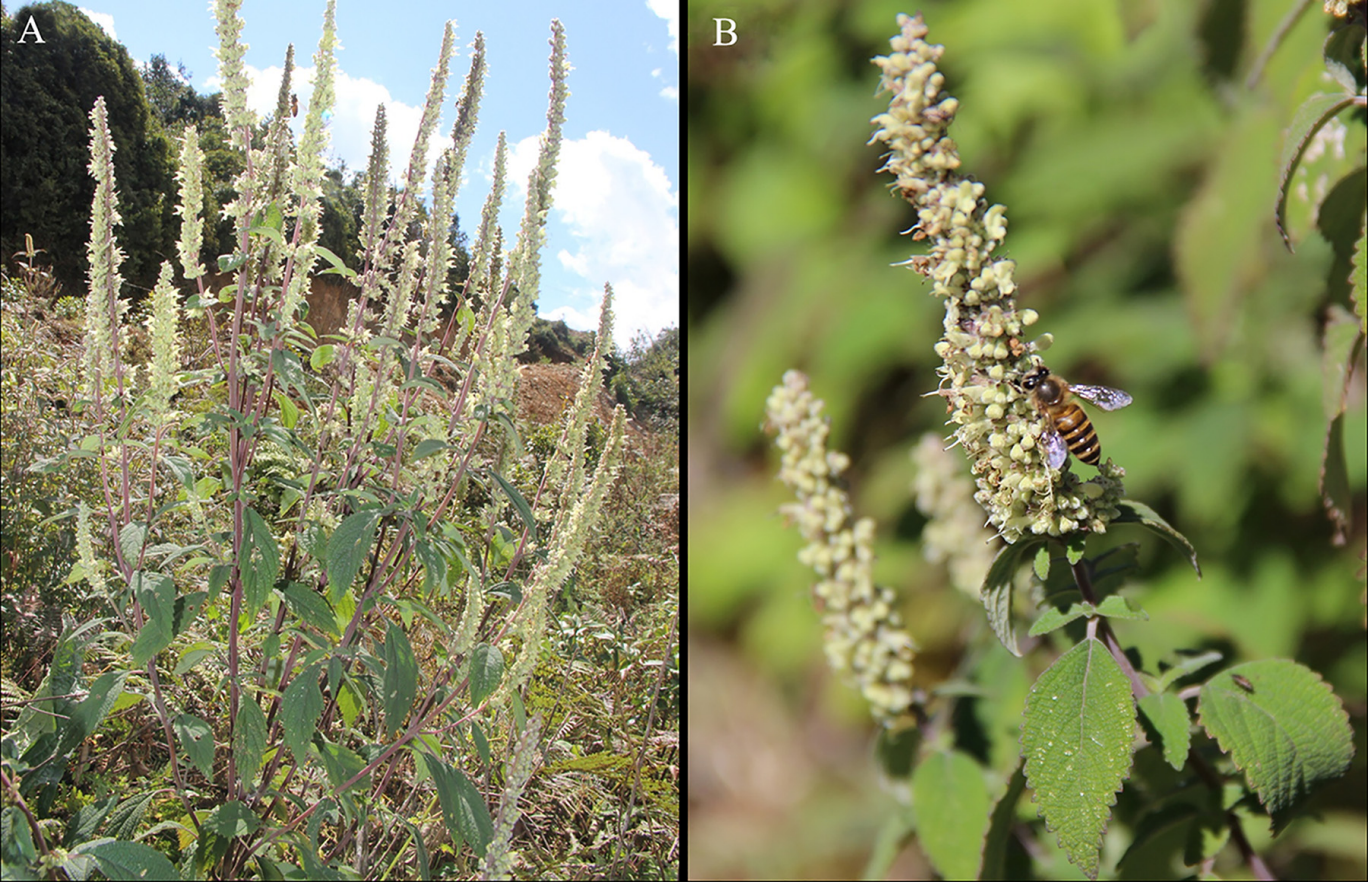
*Elsholtzia rugulosa* and its flower visitors. (A) Flowering plants of *E*. *rugulosa*. (B) *Apis cerana* feeding from flowers.

*Elsholtzia rugulosa* plants had 32.67 ± 2.68 tooth-brushy inflorescences (*n* = 30, mean ± SE) (Fig 1), each bearing 614.87 ± 8.99 flowers (*n* = 30) with a depth of 4.20 ± 0.04 mm (*n* = 30) and a style length of 5.16 ± 0.08mm (*n* = 30). The flower lifespan was 4.13 days ± 0.26 (*n* = 30), flower maturation passed in a wave across the inflorescences in c. 1 month, resulting in clumps of mature flowers at different locations during different periods. Flowers produced a small amount of nectar, the average nectar per flower was 0.48 ± 0.02μL (*n* = 32) with the average sugar concentration of 19.67% ± 0.26 (*n* = 32). The average nectar per flower on the morning was 0.48 ± 0.03μL (*n* = 15) with the average sugar concentration of 19.53% ± 0.39 (*n* = 15), and the average nectar per flower on the afternoon was 0.48 ± 0.02μL (*n* = 17) with the average sugar concentration of 19.79% ± 0.35 (*n* = 17).

### Pollinator observations and pollination experiments

During the field observation period, flowers of *E*. *rugulosa* were visited almost exclusively by the Asian honey bee, *Apis cerana*, collecting nectar, its visitation frequency was 4.34 ± 0.22 (times/h/flower) (*n* = 41). Floral buds were bagged with plastic bags, these bags were taken away when the flowers open completely, after bees visiting, these styles and anthers were removed carefully and checked. The result showed that these styles have pollens of *E*. *rugulosa*, and pollens of anthers were removed. The presence of pollen grains of *E*. *rugulosa* on the heads of the Asian honey bees and the effective contact between the head of the bees and the anthers and stigmas of the flowers during nectar collecting were confirmed. In addition, the hornet (*Vespa velutina*) and a bumblebee (*Bombus eximius*) were observed to visit occasionally the flowers of *E*. *rugulosa*, they spotted on this site, which may be attracted by other plants, and the average lengths of the protruding sections of the styles and stamens of flowers were longer than the average lengths of hornet and bumble bees’ tongues, and we visually confirmed that their heads and bodies do not made effective contact with the anthers and stigmas during visiting flowers occasionally. At the study site the few hornets and bumblebees caught in the mist net were lacking *E*. *rugulosa* pollen. Moreover, they did not attempt to drink the nectar or only briefly so. Apparently, *Apis cerana* was the only pollinator of *E*. *rugulosa* at the study site.

*Elsholtzia rugulosa* plants are self-compatible but dependent on pollinators to increase the reproductive output. The fruit set per flower on inflorescences from which Asian honey bees were excluded was significantly lower than for open-pollinated inflorescences (9.58% vs. 90.40%; *P* < 0.001). And fewer seeds were produced by bagged control flowers comparing to the open-pollinated flowers (1.38 ± 0.08 vs. 2.62 ± 0.02, *P* < 0.001). Fruit set of self-pollination was lower than that of cross-pollination (41.98% vs. 88.16%; *P*< 0.001). In addition, the mean number of seeds in fruits arising from cross-pollination was higher than in fruits arising from self-pollination (2.59 ± 0.02 vs. 1.43 ± 0.04, *P* < 0.001)

### Behavioral experiments

**Fig 2:**
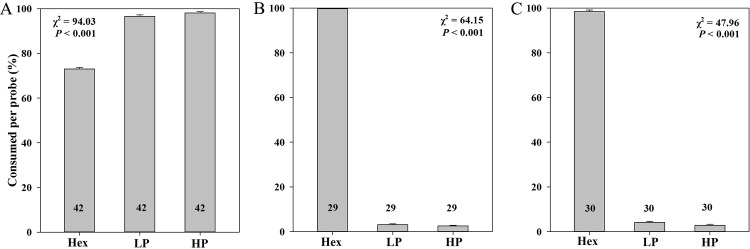
Preferences of *Apis cerana* (A), *Vespa velutina* (B) and *Bombus eximius* (C) when offered a choice among hexose (Hex), low—phenolic (LP) and high—phenolic (HP) syrups.

**Fig 3:**
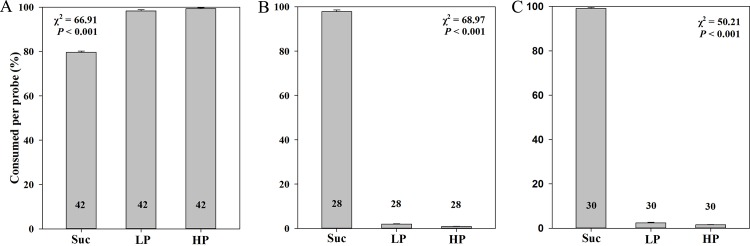
Preferences of *Apis cerana* (A), *Vespa velutina* (B) and *Bombus eximius* (C) when offered a choice among sucrose (Suc), low—phenolic (LP) and high—phenolic (HP) syrups.

Feeding trials showed that Asian honey bees significantly preferred hexose and sucrose solutions containing low or high phenolic concentrations (Figs 2 and 3) over non-phenolic solutions. However, the hornets and bumblebees strongly rejected the phenolic hexose and sucrose solutions during the feeding experiments (Figs 2 and 3). The amounts of the uptake of honey bees were 3.65 ± 0.03 (Hex), 4.83 ± 0.03 (Hex with LP), 4.91 ± 0.03 (Hex with HP), 3.98 ± 0.03 (Suc), 4.91 ± 0.03 (Suc with LP), 4.97± 0.02 (Suc with HP). The amounts of the uptake of hornets were 4.92 ± 0.03 (Hex), 0.20 ± 0.03 (Hex with LP), 0.14 ± 0.02 (Hex with HP), 4.95 ± 0.03(Suc), 0.12 ± 0.02 (Suc with LP), 0.08 ± 0.01 (Suc with HP). The amounts of the uptake of bumblebees were 4.99 ± 0.01 (Hex), 0.16 ± 0.02 (Hex with LP), 0.13 ± 0.01 (Hex with HP), 4.89 ± 0.04 (Suc), 0.10 ± 0.01 (Suc with LP), 0.04 ± 0.01 (Suc with HP). The hornets and bumblebees showed an adverse reaction to hexose and sucrose solutions with phenolics directly after probing, they shook their heads and wiped their bills rapidly after probing the sugar solutions with phenolics, which was never observed in Asian honey bees. The amounts of droplet let behind of honey bees were 1.35 ± 0.03 (Hex), 0.17 ± 0.03 (Hex with LP), 0.09 ± 0.03 (Hex with HP), 1.02 ± 0.03 (Suc), 0.09 ± 0.03 (Suc with LP), 0.03± 0.02 (Suc with HP). The amounts of droplet let behind of hornets were 0.08 ± 0.03 (Hex), 4.80 ± 0.03 (Hex with LP), 4.86 ± 0.02 (Hex with HP), 0.05 ± 0.03(Suc), 4.88 ± 0.02 (Suc with LP), 4.92 ± 0.01 (Suc with HP). The amounts of droplet let behind of bumblebees were 0.01 ± 0.01 (Hex), 4.84 ± 0.02 (Hex with LP), 4.87 ± 0.01 (Hex with HP), 0.11 ± 0.04 (Suc), 4.90 ± 0.01 (Suc with LP), 4.96 ± 0.01 (Suc with HP).

## Discussion

The primary ecological function of floral nectar is to attract and reward pollinators for transferring pollen and sweet-tasting nectar is very attractive to floral visitors. However, nectar containing phenolic compounds has an astringent taste [20, 21] and may be toxic [4, 5]. The presence of phenolics seems extremely paradoxical. Our results suggest that this paradox can be solved to a large extent if the plant’s effective pollinators are attracted by the astringent taste, while the ineffective pollinators are deterred by the bitter taste and/or toxicity. The low-phenolic or high-phenolic syrup is clearly distasteful to both hornets and bumble bees. The nectars of *E*. *rugulosa* were collected by pressing the honey stomachs of honey bees, the nectar was found to contain phenolics [5]. Honey bees do not directly feed on nectar [22] but consume the processed and more palatable honey. The flowers of *E*. *rugulosa* are effectively pollinated by Asian honey bees, as evidenced by the pollen loads they carry, by the direct contact between bee and stigmas during nectar collecting, and by the significant decrease of the fruit set and seed production in inflorescences from which Asian honey bees are excluded. Field observations of this study show that the hornets and bumble bees are occasional floral visitors of *E*. *rugulosa*, reluctant to feed on its nectar, and the longer tongues of hornets and bumble bees were unsuitable for pollination of *E*. *rugulosa*. The hornets and bumblebees might be attracted by other plants and not by *E*. *rugulosa*, which may explain why they had no pollen of *E*. *rugulosa* but the pollen of *E*. *ciliata*.

High relative bitterness of nectar from *E*. *rugulosa* suggests that the assumptive deterrent action due to the phenolic compounds in the nectar. The high concentration of buckwheat phenolic compounds (80mg phenolics per 100g syrup) in this study is the maximum dose in common honey [11, 23], and was expected to have a strong deterrence for *Apis cerana*. However, the Asian honey bees showed a significant preference for syrup with phenolics in the present study (Figs 2 and 3). Asian honey bees were frequently visiting *E*. *rugulosa* flowers and collecting its nectar in the field study. We conclude that phenolic compounds significantly stimulate the Asian honey bees to collect the nectar. The preference of the Asian honey bees for phenolic syrup is consistent with previous studies that phenolics can act as pollinator attractants [14, 15, 24–26]. In contrast, Western honey bees usually reject nectar with phenolics [6]. Pollinators may overcome phenolics in nectar through a process of coevolution [7]. It is likely that honey bees are preadapted for the astringency of nectar [19], because of the processing of the nectar into honey but the amount of processing might be variable among honey bee species.

The rejection of syrup with phenolics by hornets and bumble bees in the feeding experiments could be the result of the used sugar concentrations and sugar composition. The experiments showed that this is highly unlikely because the non-phenolic hexose and sucrose sugar solutions of the same sugar concentration and composition were freely consumed. It strongly suggests that phenolic compounds have a deterrent effect on other nectarivores as hornets and bumble bees.

Phenolic compounds should be selected for in plants that are interspersed with many other flowering plant species [7]. However, in case of *E*. *rugulosa*, fidelity is implausible as the sole explanation, since it is the main flowering plant present [15]. There should be no need to reinforce pollinator fidelity with toxic nectar because all pollinators should deposit conspecific pollen on *E*. *rugulosa* stigmas. Phenolic appears to function as an attraction signal to increase cross pollination when they move pollen from one flower to the next flower. Phenolics in nectar may also be important to the honey bee because of its antimicrobial role [6].

Phenolic compounds are transported by phloem and, therefore, also present in the nectaries and nectar. As a result phenolics in nectar could be a by-product of the resistance to herbivores of other floral parts such as buds, flowers or ovules [27]. Unfortunately, there is hardly any detailed information about the chemical composition of nectar and floral tissue of *E*. *rugulosa*. It would be particularly useful in understanding the adaptive function of the phenolic components in nectar.

## Conclusions

From the present study it is concluded that the 4-hydroxybenzoic acid in sugar water functions as attractant for its legitimate pollinators honey bees, and the hornet and bumblebee are deterred by this phenolic compound. The adaptation to phenolic nectar by *Apis cerana* delivers an evolutionary advantage to both actors, with the possibility that the phenolics are a by-product of another interaction. *Elsholtzia rugulosa* gains by having a much higher seed production and the pollinating Asian honey bee by having an exclusive and reliable food source during part of the winter season at high altitudes in SW China. It is necessary to pay attention to the evolutionary effects (including potential benefits) from phenolics and other constituents of the entire plant. The phenolic compounds may combine with other chemicals that differ in their ecological function in plant-animal interactions.
